# Difficulties translating antisense-mediated activation of Frataxin expression from cell culture to mice

**DOI:** 10.1080/15476286.2022.2043650

**Published:** 2022-03-15

**Authors:** Audrius Kilikevicius, Jun Wang, Xiulong Shen, Frank Rigo, Thahza P. Prakash, Marek Napierala, David R. Corey

**Affiliations:** a Department of Pharmacology and Biochemistry, UT Southwestern Medical Center, Dallas, Texas, United States; b Department of Biochemistry and Molecular Genetics, University of Alabama at Birmingham, 1825 University Blvd, Birmingham, Alabama, USA; c Ionis Pharmaceuticals, Medicinal Chemistry and Antisense Research, Carlsbad, California, USA; d Department of Neurology, UT Southwestern Medical Center, Dallas, Texas, USA

**Keywords:** Friedrich’s Ataxia; Frataxin; Antisense oligonucleotide; Trinucleotide repeat

## Abstract

Friedreich’s ataxia (FA) is an inherited neurodegenerative disorder caused by decreased expression of frataxin (FXN) protein. Previous studies have shown that antisense oligonucleotides (ASOs) and single-stranded silencing RNAs can be used to increase expression of frataxin in cultured patient-derived cells. In this study, we investigate the potential for oligonucleotides to increase frataxin expression in a mouse model for FA. After confirming successful *in vivo* delivery of oligonucleotides using a benchmark gapmer targeting the nuclear noncoding RNA Malat1, we tested anti-*FXN* oligonucleotides designed to function by various mechanisms. None of these strategies yielded enhanced expression of *FXN* in the model mice. Our inability to translate activation of *FXN* expression from cell culture to mice may be due to inadequate potency of our compounds or differences in the molecular mechanisms governing *FXN* gene repression and activation in FA model mice.

## Introduction

Friedreich’s ataxia (FA) is a currently incurable genetic disease caused by the expansion of the trinucleotide GAA within intron 1 of the *frataxin* (*FXN*) gene [1,2]. This mutation leads to reduced expression of *FXN* gene at the level of both RNA and protein while leaving the amino acid composition of FXN protein unchanged. This deficiency of FXN protein is the root cause of FA and compounds that restore FXN protein expression have the potential to be effective strategies for slowing the progression of disease.

The most likely cause of reduced *FXN* expression is hybridization of the expanded GAA intronic repeat within pre-mRNA to chromosomal DNA at the *FXN* locus through R-loop formation [3,4]. This R-loop promotes histone modifications in the *FXN* gene and acts as a break in transcription. While *FXN* expression is not abolished, the reduction is sufficient to cause late onset progressive disease.

Our laboratory and others have investigated antisense oligonucleotides (ASOs) and duplex RNAs (dsRNAs) that target expanded CAG trinucleotide repeats to inhibit gene expression [5–7]. *FXN* presents a different challenge because, in contrast to disease genes containing CAG repeats, the expanded repeat RNA is acting in the nucleus, the repeat is GAA rather than CAG, and the GAA repeat is within an intron rather than within a coding region.

Based on our ability to design oligonucleotides to bind CAG repeats, we hypothesized that dsRNA and ASOs designed to block the GAA repeat would prevent the formation of the R-loop with chromosomal DNA and restore *FXN* gene expression. We have previously reported that steric blocker ASOs [8–10], gapmer ASOs [11], single-stranded silencing RNAs [12] and duplex RNAs [8] that are complementary to the GAA repeat share an ability to increase *FXN* expression.

After demonstrating that activation of *FXN* gene expression could be achieved in cultured cells, the critical question was whether ASOs or dsRNAs were a viable approach to developing drugs to treat FA. There is precedent for believing that ASOs can be successful drugs inside the nuclei of cells within the central nervous system. Spinraza is an ASO designed to increase expression of a stable form of survival motor neuron 2 (SMN2) protein and act as a therapy for spinal muscular atrophy (SMA) [13]. Spinraza was approved by the FDA in 2016 and is a landmark because it demonstrated conclusively that ASOs could be active in the central nervous system and have a profoundly positive effect on the progression of a disease with high unmet need. The success of Spinraza has encouraged the belief that ASOs can function in the central nervous system and treat disease, leading us to examine the activities of anti-GAA oligonucleotides in FA model mice.

We tested steric block ASOs, gapmer ASOs, and ss-siRNAs in FA model mice. We observed no increase in *FXN* RNA or protein expression, with the exception of mice treated with the anti-GAA gapmer where we observed a decrease in *FXN* expression. These unfavourable outcomes may be due to a lack of potency or mechanistic/structural obstacles that prevented successful engagement of the ASOs with the target repeat RNA. It is also possible, however, that the mice model does not adequately mimic the subtle mechanisms that regulate expression of the *FXN* gene in human cells.

## Materials and methods

### Fxn^null^::YG8s(GAA)_>800_ mouse model and genotyping

Fxn^null^::YG8s(GAA)>800 mouse model of FA used in these studies was acquired from Jackson Laboratory (Fxntm1Mkn Tg (FXN)YG8Pook/2 J (YG8sR); stock no: 030395). These animals carry a single integrated copy of human *FXN* gene (YAC transgene) containing ~800 GAA repeats in the intron 1 of the *FXN* gene. Human *FXN* transgene is expressed on the background of null mouse *Fxn* (deletion of exon 2 of the *Fxn*). Breeding of single copy *FXN* transgenic animals was performed resulting in mice carrying both one and two copies of the FXN transgene. The q-PCR of mouse tail DNA, according to the protocol of The Jackson Laboratory (Protocol 30,021), was utilized to distinguish between animals carrying one or two copies of the transgene. The number of GAA repeats was determined for each animal using long-range PCR as described in [14].

All the mice were housed in an environment of 12 h light/dark cycle, temperature of 25 ± 2°C, 55% humidity, and ad libitum standard diet and water. All animal studies were performed in accordance with standard regulations and were approved by the Institutional Animal Care and Use Committee, University of Alabama at Birmingham IACUC-20579.

### Intracerebroventricular (ICV) injection of oligonucleotides into neonatal mice

We tested several ASOs by delivering them through a single intracerebroventricular (ICV) injection into the brain of day 0–1 mice [15]. Briefly, pups were put on ice for anaesthesia, and the fully anaesthetized pups (confirmed by lack of movement and also by gently squeezing a paw to monitor for lack of movement) were moved under the dissecting microscope to visualize the sagittal suture, lambda and bregma. After skin disinfection with 2.5% betadine, an injection site was located approximately 0.8–1 mm lateral from the sagittal suture, halfway between lambda and bregma. The needle was inserted at the marked injection site to a depth of approximately 3 mm, and a volume of 3 µl was administered into each ventricle. After injections into both hemispheres, the injected pups were returned to the biological mother after they recovered normal skin colour and movement. Mice were sacrificed at day 14. The brain, cerebellum and spinal cord were collected, flash frozen in liquid nitrogen and stored at −80°C for later RNA and protein analysis.

### ICV injection of oligonucleotides into adult mice

The YG8sR mice of 8 weeks old were injected with oligonucleotides in 10 µl by ICV into the right lateral ventricle. Briefly, mouse was placed in stereotaxic apparatuses (ASI Instruments, Small Animal Stereotaxic System, SAS-4100) such that the 18^°^ ear bars were in the ear canals and the incisors were in the tooth bar of the mouse adapter. Surgical planes of anaesthesia were maintained with 2% Isoflurane by a nose cone. A 1–1.5 cm slightly off-centre incision was made in the scalp. The needle was positioned over bregma and the coordinates 0.3 mm anterior and 1 mm to the right medial/lateral were used. The needle tip was advanced through the skull until the lumen of the needle passes the skull surface and advanced to the desired coordinate, which was 3.0 mm dorsal/ventral. The proper amount of injection solution, 10 µL, was injected by hand at an injection rate of approximately 1 µL/second. The incision was sutured and closed using one horizontal mattress stitch with 5-O Ethilon suture. The animals were then allowed to recover from the anaesthesia in their home cage. The brain, cerebellum and spinal cord were collected 2 weeks after injection, flash frozen in liquid nitrogen and stored at −80°C for later RNA and protein analysis.

### Evaluation of targeted genes expression at RNA and protein levels

To determine the impact of injected oligonucleotides on genes expression, control as well as experimental brain samples were divided into two pieces and snap frozen for further analyses. One piece served for the relative quantification of RNA level, another was used to determine the effect at protein level.

The total RNA was purified using RNeasy® Mini Kit (Qiagen, Cat.#74,106) following the manufacturer’s purification protocol from animal tissues. The DNA from the RNA samples was removed using on-column DNase digestion (Qiagen, Cat.#79,256). 1 μg of purified total RNA was converted to cDNA using the High Capacity cDNA Reverse Transcription Kit with random primers (ABI, Cat#4,368,813). To quantify the relative expression level of *FXN* or *Malat1,* we used the 50 ng of cDNA per qPCR reaction (BIO-RAD, Cat.# 1,725,124) containing specific primer sets for the genes of interest or reference transcripts (Table S1). The *FXN* primer set amplifies 106 nt length product specifically spanning the exon3 and exon4 junction.

We used several reference genes with different Ct values to prevent misleading results by changes in reference gene rather than *FXN* expression after oligonucleotide treatment. Similar results from the main text normalized to different reference genes are provided as supplementary data.

Proteins from the brain tissue were extracted using RIPA lysis buffer (150 mM NaCl, 50 mM Tris-HCl, 1% NP-40, 0.25% Na-deoxycholate, 1 mM EDTA) supplemented with 1x protease inhibitor cocktail (Roche, Cat.#11,697,498,001). In brief, tissue was homogenized in RIPA buffer and kept at –80°C overnight. Defrosted samples were centrifuged at maximum speed, and the supernatant was used for Western-blot analysis as previously described [9]. Primary antibodies were used at the indicated dilution ratio: anti-Frataxin (1:20,000, Millipore, MABN2313), anti-ß-tubulin (1:5000, Sigma-Aldrich, T5201) and anti-ß-Actin (1:20,000, Sigma, A5441).

## Results

### Mouse model

We used Fxn^null^::YG8s>800 model mice in these studies [16,17]. These mice carry a single integrated copy of the human *FXN* gene with ~800 GAA repeats in intron 1. The repeat had expanded to ~800 after creation of the mouse model, presumably because of slippage and repair over generations. Repeat numbers are typically between 600 and 900 in patients [1].

The human transgene is expressed on a background of null mouse *Fxn* (deletion of exon 2), thus demonstrating that human *FXN* is properly expressed, processed and imported into mitochondria to function in iron-sulphur cluster biosynthesis processes. Since the YG8sR mice harbour entire human *FXN* gene and a large GAA tract, they represent, at present time, the best FA model to test therapeutic approaches aimed at expanded GAA repeats. Other FRDA models harbour significantly shorter GAAs [18] or rely on frataxin downregulation via non-GAA dependent approaches (shRNA conditional knock-out [19];). Frataxin expression in YG8sR mice is decreased due to the chromatin silencing [20–22]. Downregulation of the *FXN* could be partially rescued by different approaches including treatment with histone deacetylase inhibitors or Nrf2 pathway activators [23,24]. On the other hand, behavioural phenotype of YG8sR animals is rather mild and of late onset, thus much more challenging to rescue, especially in short-term studies [17].

We examined mice that were both hemi- and homo-zygous for expression of the human transgene. Consistent with the presence of two copies rather than one copy, the homozygous mice had approximately two times more expression than the hemizygous mice (Supplemental Figure S1). Oligonucleotides were delivered by intracerebroventricular (ICV) injection into either neonatal or adult mice. Hemizygous mice, which have only one transgene expressing human *FXN*, were used because we reasoned that any increase in gene expression would be most visible in mice with lower basal levels of *FXN*.

### Validation of in vivo dosing

To validate our protocol for dosing mice with ASOs, we evaluated the efficacy of an ASO gapmer (Table 1) that targets the nuclear noncoding RNA *Malat1. Malat1* is a favourable target for validating delivery protocols that use ASOs. Like the expanded GAA repeat within *FXN* intronic RNA, *Malat1* RNA is predominantly localized to the nucleus. In addition, the anti-*Malat1* ASO is a robust gene silencing agent that is a reliable benchmark for evaluating gene knock-down in target tissues. *Malat1* has no clear physiologic function in neonatal and adult mice and knocking it down should have no detrimental effects.

**Table 1.** Oligonucleotides used in these studies. TABLE 1 IS NOT IN COLOR. IF YOU WISH TO HAVE THIS TABLE IN B/W, LET US KNOW AND WE WILL REDO IT

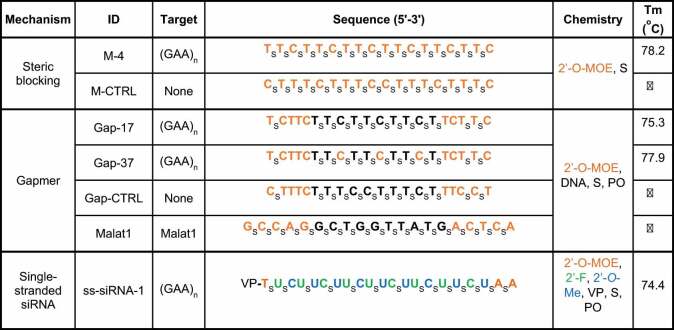

**Figure 1.**
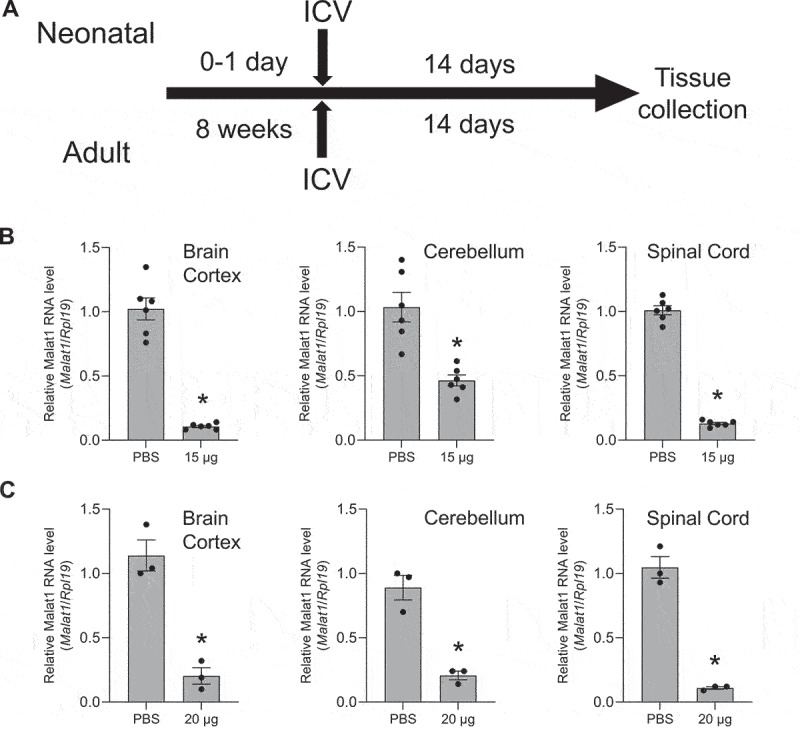
***In vivo* study design and validation of ICV injection protocol**. (a) Timeline for introducing ASOs and ss-siRNAs *in vivo* and subsequent collection of tissue. Top, timeline for neonatal mice. Bottom, timeline for adult mice. (b,c) Validation of methodology using anti-*Malat1* ASO to determine knockdown of *Malat1* nuclear noncoding RNA in brain cortex, cerebellum, and spinal cord in (b) neonatal and (C) adult mice. qPCR data were normalized against the *Rpl19* gene. T-test * – p < 0.01.

We examined knockdowns in both neonatal and adult mice using tissue collected 14 days after ICV injection (Figure 1a). In both cases, we observed efficient knockdown of *Malat1* RNA in the cortex, cerebellum, and spinal cord relative to control mice injected with saline solution (Figure 1b,c). No toxicity was observed in the animals at the doses used in these experiments. These data confirmed that our protocols were effective in allowing ASOs to enter tissue and engage with a nuclear RNA target in the CNS to modulate gene expression.

### Effect of steric block ASOs

Steric blocking ASOs are designed to recognize a target nucleic acid sequence and block its ability to form other interactions without inducing degradation of the target [25]. We have previously reported using patient-derived cultured cells that steric blocking ASOs that target the GAA repeat within the nascent RNA can reverse R-loop formation and up-regulate *FXN* gene expression [8–10]. The most likely mechanism explaining this activation is through blocking the association of the expanded GAA repeat to its complementary sequence within chromosomal DNA.

**Figure 2.**
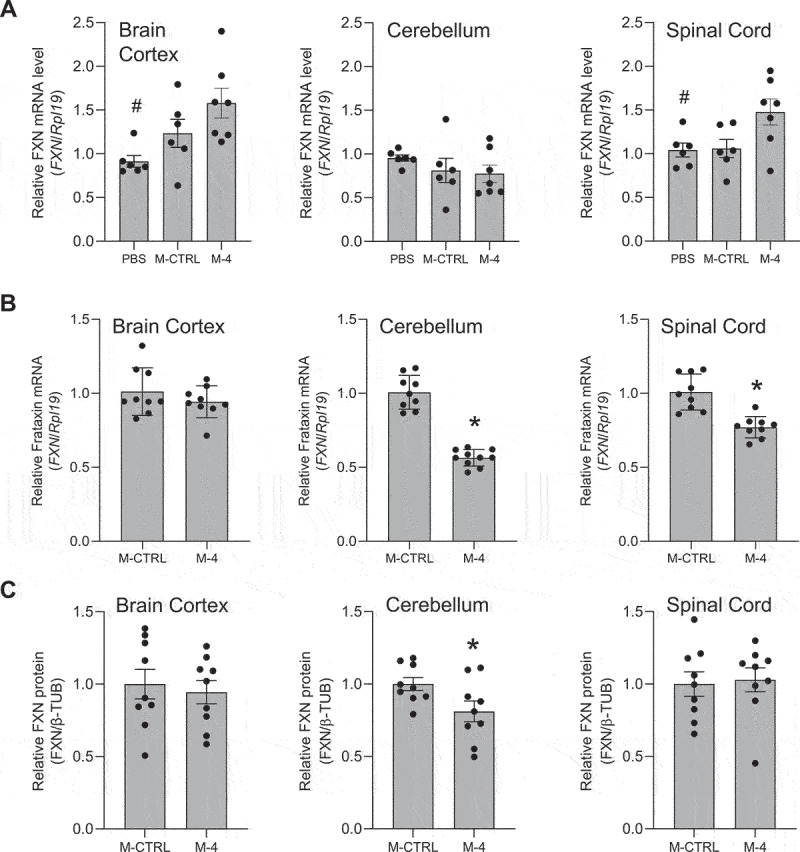
**Effect of *in vivo* delivery of a steric block ASO M-4 on *FXN* mRNA and protein levels**. Effect on *FXN* RNA levels in (a) neonatal and (b) adult mice. qPCR data were normalized against the *Rpl19* gene. Normalization against the *Gapdh* and *Hprt1* genes is shown in Supplementary Figure S2. (c) Quantitation of western analysis showing the effect of ASO administration on FXN protein levels in adult mice. Western blots are shown in Supplementary Figure S2. AVG ± SEM. One-way ANOVA (Tukey’s post-hoc) # – p < 0.05 (PBS *vs*. M-4); T-test * – p < 0.05.

**Figure 3.**
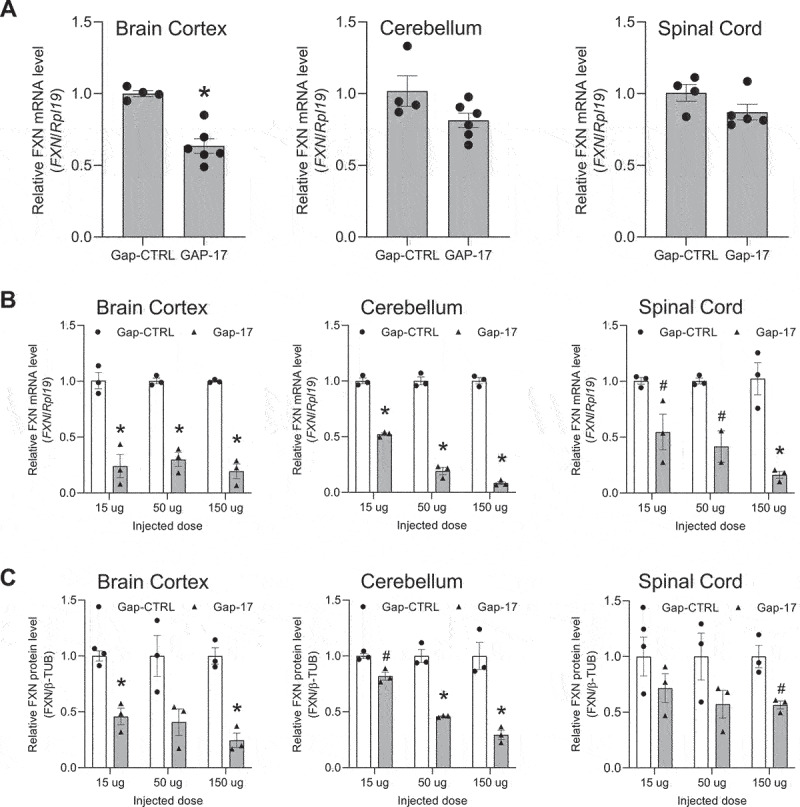
**Effect of *in viv*o delivery of gapmer ASO Gap-17 on *FXN* mRNA and protein levels**. Effect on *FXN* RNA levels in (a) neonatal and (b) adult mice. qPCR data were normalized against the *Rpl19* gene. Normalization against the *Gapdh* and *Hprt1* genes is shown in Supplementary Figure S3. (c) Quantitation of western analysis showing the effect on FXN protein levels in adult mice. Western blots are shown in Supplementary Figure S3. AVG ± SEM. T-test # – p < 0.05, * – p < 0.01.

**Figure 4.**
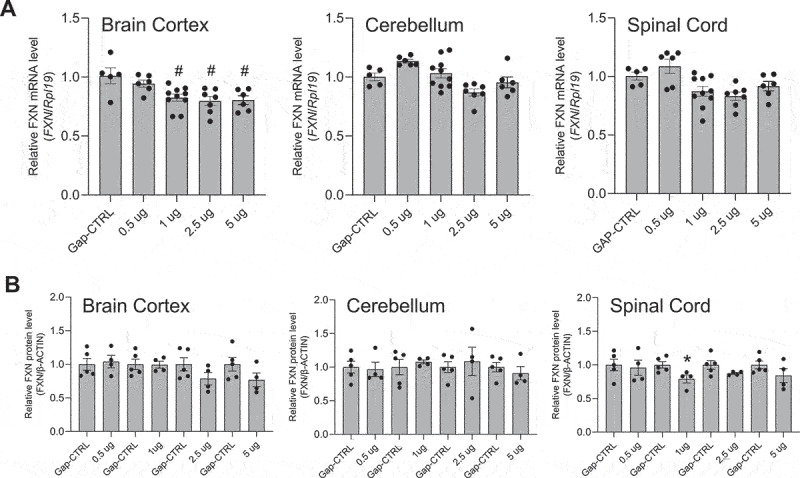
**Effect of *in vivo* delivery of ASO Gap-37 based on a gapmer design but modified to disable the potential for RNAse H cleavage**. (a) Effect on *FXN* RNA levels in neonatal mice. qPCR data were normalized against the *Rpl19* gene. Normalization against the *Gapdh* and *Hprt1* genes is shown in Supplementary Figure S4. (b) Quantitation of western analysis showing the effect on FXN protein levels in neonatal mice. Western blots are shown in Supplementary Figure S4. AVG ± SEM. One-way ANOVA (Tukey’s post-hoc) # – p < 0.05 (*vs*. Gap-CTRL); T-test * – p < 0.05.

**Figure 5.**
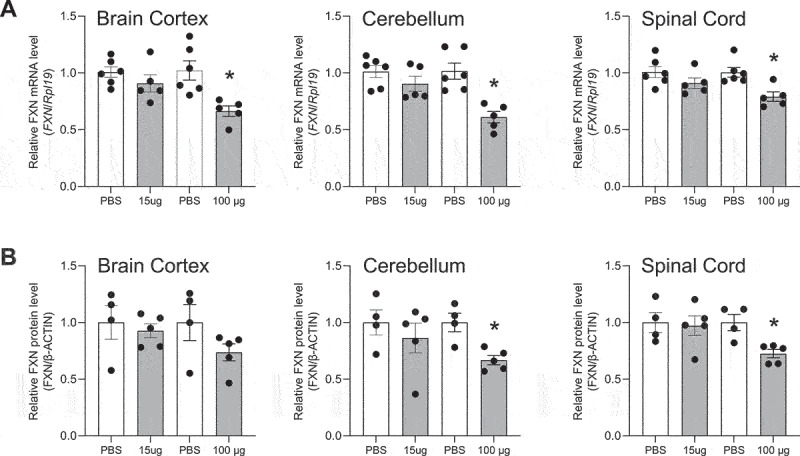
**Effect of *in vivo* delivery of ss-siRNA-1 on *FXN* mRNA and protein levels**. (a) Effect on *FXN* RNA levels in neonatal mice. qPCR data were normalized against the *Rpl19* gene. Normalization against the *Gapdh* and H*prt1* genes is shown in Supplementary Figure S5. (b) Quantitation of western analysis showing the effect on FXN protein levels in neonatal mice. Western blots are shown in Supplementary Figure S5. AVG ± SEM. T-test * – p < 0.01.

We tested steric blocking ASO M-4, an 18 base entirely methoxyethyl (MOE)-substituted phosphorothioate oligonucleotide (Table 1). Conclusions from qPCR measurement of RNA levels can vary depending on which reference gene is chosen for analysis (Figures 1–5). We used the *RPL19* gene as the primary standard, but we also included data analysing expression of the *Gapdh* and *Hprt1* genes to confirm our conclusions for ASO M-4 and the other oligonucleotides used in this study (Supplementary Figures S2-S5). ASO M-4 was administered by ICV injection into neonatal (30 µg) (Figure 2a) or adult mice (500 µg) (Figure 2b) (Supplemental Figure S2). Effects on *FXN* expression were evaluated relative to a control PS-MOE oligonucleotide, M-CRTL. For neonatal mice, we also made comparisons to saline-treated animals.

We observed no significant increase in *FXN* expression in the cortex, cerebellum, or spinal cord of neonatal mice relative to controls (Figure 2a). In adult mice, we observed significant decreases in *FXN* RNA levels in the cerebellum and spinal cord (Figure 2b). There was no change in FXN protein levels as measured by western analysis, except in cerebellum where decreasing expression was observed corresponding to significant downregulation at mRNA level (Figure 2c) (Supplemental Figure S2).

### Effect of gapmer ASOs

Gapmer oligonucleotides consist of a central DNA region that can recruit RNase H flanked by nucleotides containing chemical modifications that increase affinity to complementary sequences [25]. Gapmers are widely used in therapeutic development because their design permits cleavage of target transcripts and more potent reduction of gene expression.

We had previously hypothesized that gapmers complementary to the intronic GAA repeat would induce cleavage of the repeat, thereby improving the potency of anti-GAA ASOs and the likelihood of successful *in vivo* activation of *FXN* expression. Our initial cell culture data showed a 10-fold increase in the potency of *FXN* gene activation in patient-derived cells, and also revealed that high concentrations of gapmer cause a decrease in *FXN* expression [11]. This decrease in expression is consistent with other reports that gapmers targeting intronic regions can result in inhibition of parent gene expression [26,27] and suggests that there may be a narrow dosage window for achieving *FXN* gene activation using gapmers.

We administered complementary gapmer Gap-17 to neonatal and adult mice. Relative to control gapmer Gap-CRTL, we observed that Gap-17 caused a dose-dependent decrease in *FXN* gene expression in the cortex, cerebellum, and spinal cord of neonatal mice (Figure 3a), with statistical significance achieved in the cortex. In adult mice, administration at 15, 50 or 150 µg led to significant decreases in *FXN* in all tissues assayed (Figure 3b). Protein expression also decreased (Figure 3c, Supplementary Figure S3). Higher doses were toxic to the mice. These results suggest that GAP-17 was engaging with target and modulating gene expression but was not functioning to activate *FXN* expression.

### Effect of a modified RNAse H ‘inactivated’ gapmer ASO

Based on previous mechanistic insights from other laboratories examining the effects of gapmer-targeted ASOs [26,27], we reasoned that the cleavage of the intronic RNA might be responsible for decreased expression of the *FXN* gene. These previous experiments had shown that gapmers that target introns not only lead to cleavage of the introns but also reduce expression of the parent genes. We hypothesized that chemical modifications that ‘inactivate’ the gapmer to cleavage might yield an oligonucleotide that retains the favourable binding properties inherent in the gapmer design while removing the detrimental potential for intronic cleavage. Gapmer GAP-37 was inactivated towards RNase H by placing three MOE RNA modifications within the central DNA gap region (Table 1).

We observed that inactivated gapmer GAP-37 behaved similar to previously tested steric blocking ASO M-4 in adult mice (Figure 4, Supplemental Figure S4). We observed no consistent increase in RNA levels in neonatal when dosed with increasing amounts of Gap-37 (Figure 4a). Protein levels were unchanged in the cortex, cerebellum, or spinal cord or showed a slight decrease (Figure 4b, Supplementary Figure S4). These data showing that the introduction of MOE bases prevents lowering of *FXN* expression support the hypothesis that fully active GAP-17 was binding to the target GAA intronic region and was directly responsible for the decrease in *FXN* expression.

### Effect of an ss-siRNA

Single-stranded silencing RNAs (ss-siRNAs) are oligonucleotides that share features of traditional single-stranded ASOs and duplex RNAs. These RNAs are chemically modified to stabilize them against degradation by nucleases while permitting them to enter the RNA-induced silencing complex and act as gene silencing agents without the need for hybridization with a passenger strand to form a duplex [28–30]. The potential advantage of ss-RNAs is that they may combine the best of both antisense modalities – the better uptake in the central nervous system of single-stranded oligonucleotides relative to duplex RNAs and the potential for improved potency through interactions with the cellular RNAi protein machinery.

We administered anti-GAA ss-siRNA-1 (Table 1) to neo-natal mice by ICV injection at 15 µg or 100 µg doses. We observed no increase in the expression levels of *FXN* RNA (Figure 5a) or protein (Figure 5b) (Supplementary Figure 5) in cortex, cerebellum, or spinal cord relative to control mice treated with saline solution. At the highest dose, *FXN* expression showed a slight decline.

## Discussion

Our previous studies had demonstrated activation of frataxin expression [8–11,25]. Activation was observed in both patient-derived fibroblast cells and in induced pluripotent stem cell-derived neuronal progenitor cells with a variety of different synthetic nucleic acids including steric block ASOs [8–10], ss-siRNAs [12], duplex RNAs [8,9], and gapmer ASOs [11]. We now describe *in vivo* data using a similar range of compounds and report that we have not observed gene activation.

What reasons might explain the lack of success *in vivo*? In cell culture, our compounds were delivered into cells in complex with cationic lipid [8–11] or by electroporation [9,11]. For *in vivo* delivery, compounds were dissolved in buffered saline alone. One possibility, therefore, is that our compounds do not enter cells *in vivo*.

While the lack of uptake of individual compounds can never be discounted as an obstacle, we note that there have been several robust demonstrations of similar nucleic acid compounds showing powerful target-directed effects in the central nervous system of mice and humans [25]. In our hands, the positive control anti-*Malat1* gapmer was active, suggesting that protocols for in vivo delivery were effective and robust. Specific to anti-*FXN* oligonucleotides, the gapmer oligonucleotides tested in our studies tended to decrease *FXN* expression. This is consistent with observations that gapmers targeting intronic RNA cause a decrease in expression of the target gene [26,27]. We conclude that, while lack of uptake might have contributed to the lack of activity, it is not likely to be the only explanation.

Another explanation is the lack of potency for the compounds that rely on steric blocking mechanisms. Typically, when an ASO is targeting mRNA, there are hundreds of potential target sequences and, therefore, hundreds of potentially active ASOs. Some ASOs will be more active than others and, as with any other drug discovery effort, the most potent compounds will be prioritized for testing. In this case, however, we were constrained by the need to target the expanded GAA repeat. The options for finding highly active ASOs were limited by the repetitive nature of the poly GAA region.

We had previously observed that anti-GAA gapmers were more potent than steric blocking ASOs in cell culture testing [11]. This potency, however, was accompanied by reduced *FXN* expression and toxicity when used at higher concentrations. Our cell culture data suggest that the effective window for activation of *FXN* expression by gapmers is small and was not achieved in our *in vivo* experiments.

A final explanation is that the mechanism of R-loop formation and oligonucleotide recognition that we hypothesize to be responsible for the gene activation in human cells is subtle. The mechanism is not a straightforward nor is it as well characterized as the action of gapmer ASOs that target mRNA or ASOs that re-direct splicing [25]. It is possible, therefore, that the mechanisms governing the interplay of expanded GAA DNA and RNA in mice are different from human cells, reducing the potential for ASOs to have favourable effects.

Importantly, human *FXN* transgene has been randomly integrated into mouse genome (on chromosome 16) [17]; thus, locus context, transcriptional activity of neighbouring genes and potentially higher order chromatin organization of this locus may contribute to the lack of ASOs efficacy in mice. While ‘backing off’ and moving from *in vivo* experiments to experiments with cultured cells is often unappealing, *ex vivo* experiments model mouse cells might be necessary to gain more insight into the value of the mouse model for test anti-GAA ASOs.

There is an urgent need to develop treatments for FA. The expanded trinucleotide repeat is the root cause of the disease and is common to almost all FA patients. The trinucleotide repeat remains the potential ‘Achilles’ heel’ for disease progression and a target for drug development.

While the outcomes presented here are disappointing, we hope that the lessons learned will enable a more successful discovery of oligonucleotide drugs in the future. The mechanism of reduced frataxin expression is complicated. The formation, persistence, and exact mechanism of R-loop formation are not well understood. Recognition of the repeat RNA by oligonucleotides is also likely to be a complex process. Our negative results should be interpreted as a lesson for future research, not a definitive finding that a therapy cannot be achieved by targeting the mutant GAA repeat. We have not achieved successful translation from *in vitro* to *in vivo*, but it remains possible that the discovery of more potent compounds in combination with a better understanding of *FXN* expression in FA animal models and reconsidering more complex disease mechanisms beneath R-loop formation may lead to great success in the future.

## Supplementary Material

Supplemental Material

Supplemental Material

## Data Availability

The authors confirm that the data supporting the findings of this study are available within the article [and/or] its supplementary materials.
