# Supplementary material for: Difficulties translating antisense-mediated activation of Frataxin expression from cell culture to mice
Source: RNA Biol. 2022 Mar 15;19(1):364–72. doi: 10.1080/15476286.2022.2043650 (PMC8928816; doi:10.1080/15476286.2022.2043650)
Supplement: Supplemental Material [file KRNB_A_2043650_SM5777.pptx]

## Slide 1
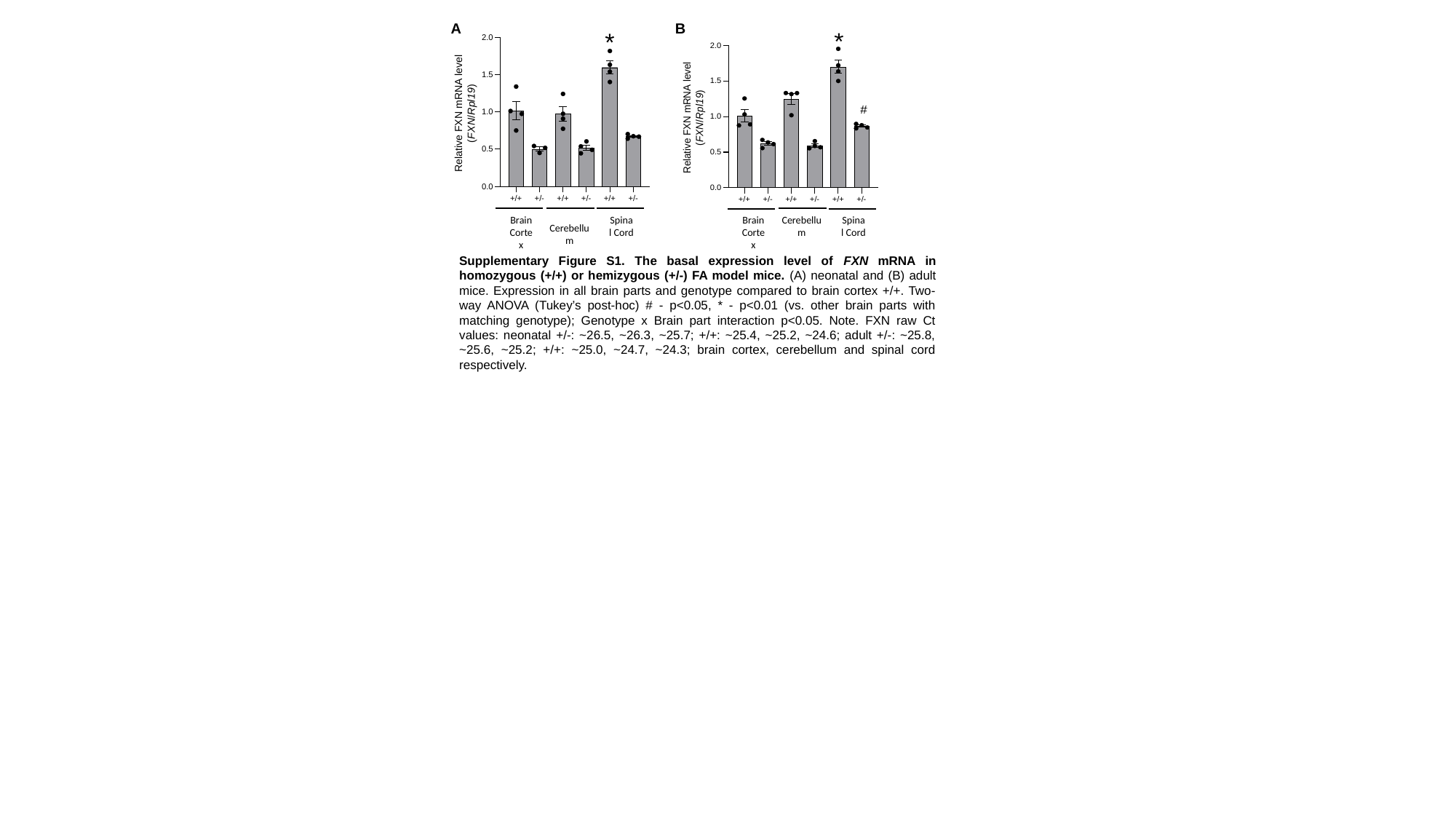

A
B
Brain Cortex
Spinal Cord
Brain Cortex
Cerebellum
Spinal Cord
Cerebellum
Supplementary Figure S1. The basal expression level of FXN mRNA in homozygous (+/+) or hemizygous (+/-) FA model mice. (A) neonatal and (B) adult mice. Expression in all brain parts and genotype compared to brain cortex +/+. Two-way ANOVA (Tukey’s post-hoc) # - p<0.05, * - p<0.01 (vs. other brain parts with matching genotype); Genotype x Brain part interaction p<0.05. Note. FXN raw Ct values: neonatal +/-: ~26.5, ~26.3, ~25.7; +/+: ~25.4, ~25.2, ~24.6; adult +/-: ~25.8, ~25.6, ~25.2; +/+: ~25.0, ~24.7, ~24.3; brain cortex, cerebellum and spinal cord respectively.

## Slide 2
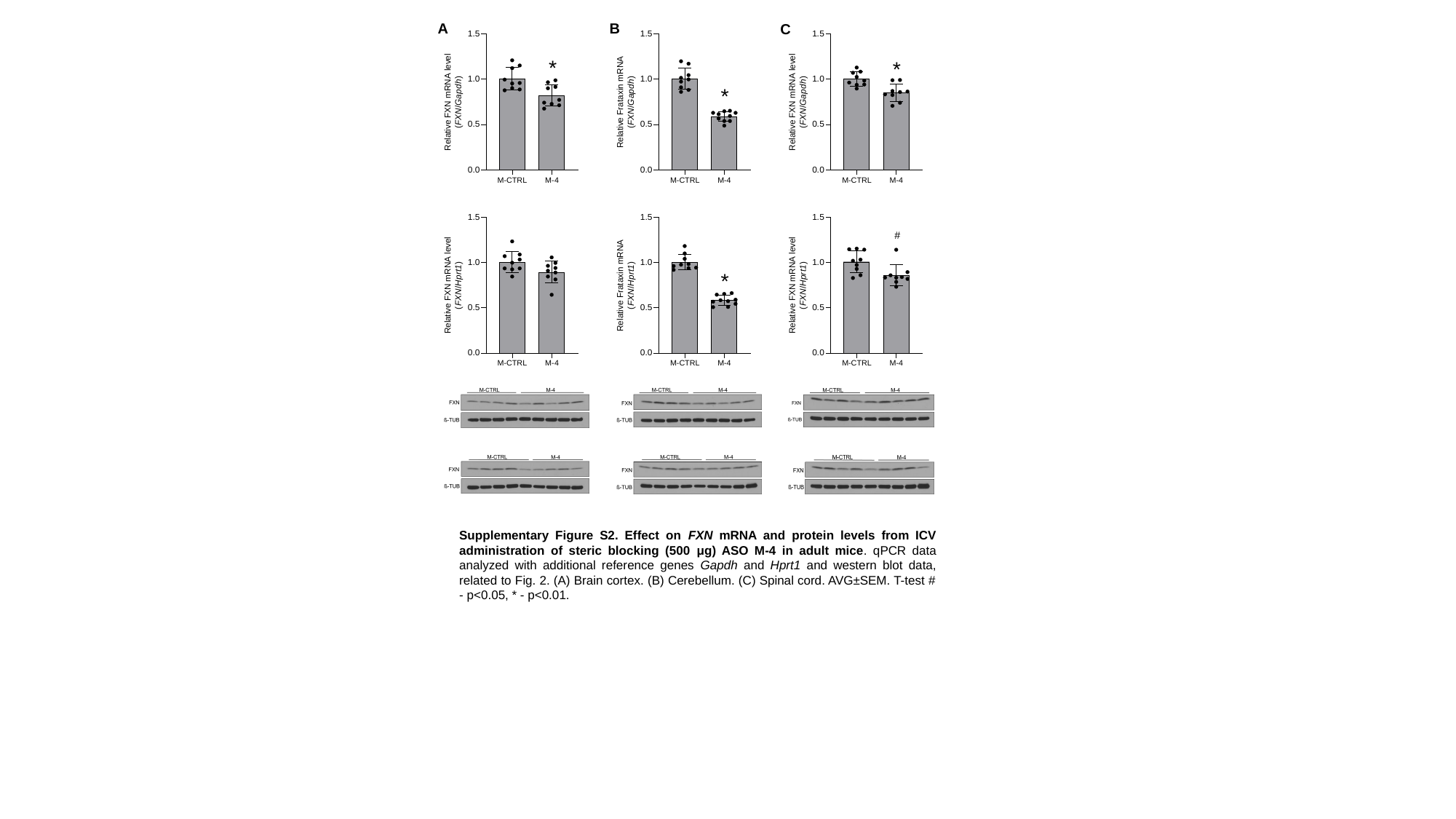

A
B
C
Supplementary Figure S2. Effect on FXN mRNA and protein levels from ICV administration of steric blocking (500 μg) ASO M-4 in adult mice. qPCR data analyzed with additional reference genes Gapdh and Hprt1 and western blot data, related to Fig. 2. (A) Brain cortex. (B) Cerebellum. (C) Spinal cord. AVG±SEM. T-test # - p<0.05, * - p<0.01.

## Slide 3
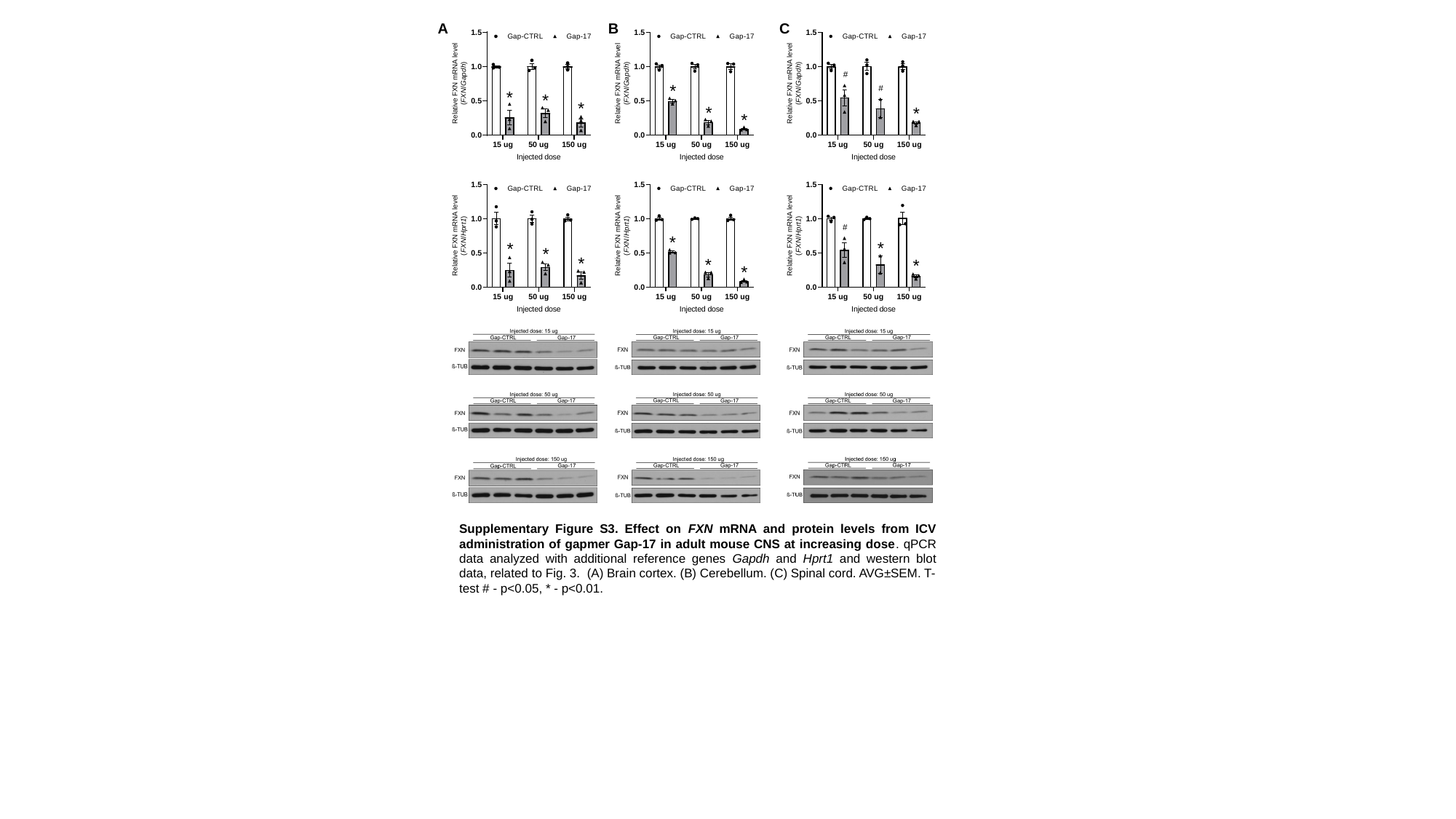

A
B
C
Supplementary Figure S3. Effect on FXN mRNA and protein levels from ICV administration of gapmer Gap-17 in adult mouse CNS at increasing dose. qPCR data analyzed with additional reference genes Gapdh and Hprt1 and western blot data, related to Fig. 3. (A) Brain cortex. (B) Cerebellum. (C) Spinal cord. AVG±SEM. T-test # - p<0.05, * - p<0.01.

## Slide 4
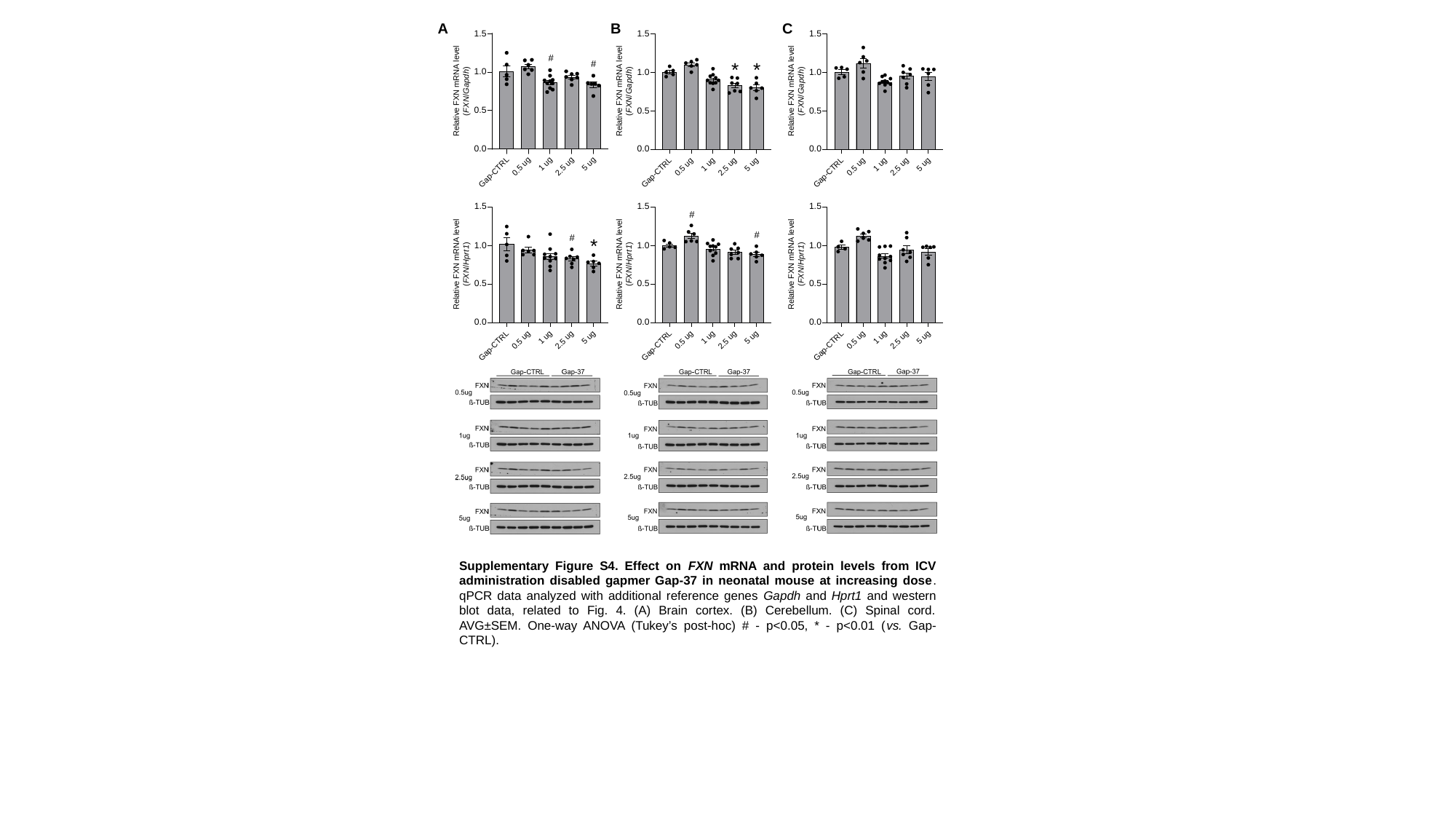

A
B
C
Supplementary Figure S4. Effect on FXN mRNA and protein levels from ICV administration disabled gapmer Gap-37 in neonatal mouse at increasing dose. qPCR data analyzed with additional reference genes Gapdh and Hprt1 and western blot data, related to Fig. 4. (A) Brain cortex. (B) Cerebellum. (C) Spinal cord. AVG±SEM. One-way ANOVA (Tukey’s post-hoc) # - p<0.05, * - p<0.01 (vs. Gap-CTRL).

## Slide 5
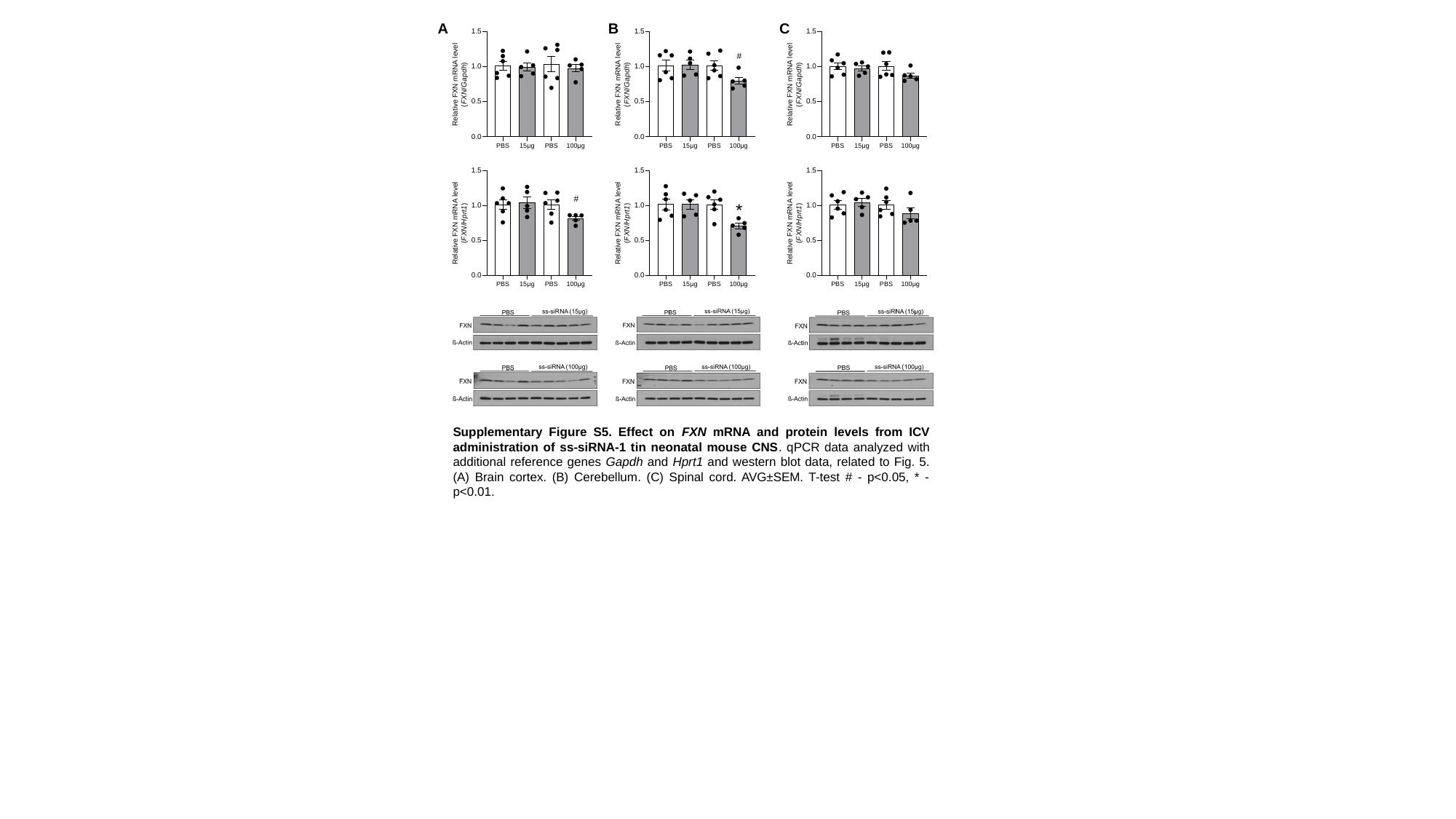

A
B
C
Supplementary Figure S5. Effect on FXN mRNA and protein levels from ICV administration of ss-siRNA-1 tin neonatal mouse CNS. qPCR data analyzed with additional reference genes Gapdh and Hprt1 and western blot data, related to Fig. 5. (A) Brain cortex. (B) Cerebellum. (C) Spinal cord. AVG±SEM. T-test # - p<0.05, * - p<0.01.
