# Supplementary material for: Difficulties translating antisense-mediated activation of Frataxin expression from cell culture to mice
Source: RNA Biol. 2022 Mar 15;19(1):364–72. doi: 10.1080/15476286.2022.2043650 (PMC8928816; doi:10.1080/15476286.2022.2043650)
Supplement: Supplemental Material [file KRNB_A_2043650_SM5740.docx]

**Supplementary Table S1.** Primers use for RNA amplification.

| **Gene** | **Primers sequence 5’ to 3’** |
| --- | --- |
| *FXN* | F – AAGCCATACACGTTTGAGGACTA  R – TTGGCGTCTGCTTGTTGATCA |
| *Malat1* | F – AGCTTTTGAGGGCTGACTGC  R – CCATTCATTCCCCTCTGAGC |
| *Rpl19* | F – GTATGCTCAGGCTACAGAAGAG  R – GAGTTGGCATTGGCGATTT |
| *Hprt1* | F – AGTCCCAGCGTCGTGATTAG  R – TTTCCAAATCCTCGGCATAATGA |
| *Gapdh* | F – AGGTCGGTGTGAACGGATTTG  R – TGTAGACCATGTAGTTGAGGTCA |
